# First outline and baseline data of a randomized, controlled multicenter trial to evaluate the health economic impact of home telemonitoring in chronic heart failure – CardioBBEAT

**DOI:** 10.1186/s13063-015-0886-8

**Published:** 2015-08-11

**Authors:** Reiner Hofmann, Heinz Völler, Klaus Nagels, Dominik Bindl, Eik Vettorazzi, Ronny Dittmar, Walter Wohlgemuth, Till Neumann, Stefan Störk, Oliver Bruder, Karl Wegscheider, Eckhard Nagel, Eckart Fleck, on behalf of the CardioBBEAT Investigators

**Affiliations:** Institute for Healthcare Management and Health Sciences, University of Bayreuth, Prieserstraße 2, 95444 Bayreuth, Germany; Rehabilitation Center for Internal Medicine, Klinik am See, Seebad 84, 15562 Rüdersdorf, Germany; Center of Rehabilitation Research, University of Potsdam, Am Neuen Palais 10, 14469 Potsdam, Germany; Department of Medical Biometry and Epidemiology, University Medical Center Hamburg-Eppendorf, Martinistraße 52, 20246 Hamburg, Germany; Professional Board of German Surgeons, Luisenstraße 58/59, 10117 Berlin, Germany; Radiology, University Medical Center of Regensburg, Franz-Josef-Strauß-Allee 11, 93053 Regensburg, Germany; Clinic for Cardiology, University Hospital Essen, Hufelandstraße 55, 45147 Essen, Germany; Comprehensive Heart Failure Center Würzburg and Department of Internal Medicine I, University of Würzburg, Straubmühlweg 2a, 97078 Würzburg, Germany; Contilia Heart and Vascular Center, Department of Cardiology and Angiology, Elisabeth Hospital Essen, Klara-Kopp-Weg 1, 45138 Essen, Germany; Department of Medicine/Cardiology, German Heart Institute Berlin, Augustenburger Platz 1, 13353 Berlin, Germany

**Keywords:** Home telemonitoring, Chronic heart failure (CHF), Incremental Cost-Effectiveness Ratio (ICER), Mortality, Telemedicine, Health economics

## Abstract

**Background:**

Evidence that home telemonitoring for patients with chronic heart failure (CHF) offers clinical benefit over usual care is controversial as is evidence of a health economic advantage.

**Methods:**

Between January 2010 and June 2013, patients with a confirmed diagnosis of CHF were enrolled and randomly assigned to 2 study groups comprising usual care with and without an interactive bi-directional remote monitoring system (Motiva®). The primary endpoint in CardioBBEAT is the Incremental Cost-Effectiveness Ratio (ICER) established by the groups’ difference in total cost and in the combined clinical endpoint “days alive and not in hospital nor inpatient care per potential days in study” within the follow-up of 12 months.

**Results:**

A total of 621 predominantly male patients were enrolled, whereof 302 patients were assigned to the intervention group and 319 to the control group. Ischemic cardiomyopathy was the leading cause of heart failure. Despite randomization, subjects of the control group were more often in NYHA functional class III–IV, and exhibited peripheral edema and renal dysfunction more often. Additionally, the control and intervention groups differed in heart rhythm disorders. No differences existed regarding risk factor profile, comorbidities, echocardiographic parameters, especially left ventricular and diastolic diameter and ejection fraction, as well as functional test results, medication and quality of life. While the observed baseline differences may well be a play of chance, they are of clinical relevance. Therefore, the statistical analysis plan was extended to include adjusted analyses with respect to the baseline imbalances.

**Conclusions:**

CardioBBEAT provides prospective outcome data on both, clinical and health economic impact of home telemonitoring in CHF. The study differs by the use of a high evidence level randomized controlled trial (RCT) design along with actual cost data obtained from health insurance companies. Its results are conducive to informed political and economic decision-making with regard to home telemonitoring solutions as an option for health care. Overall, it contributes to developing advanced health economic evaluation instruments to be deployed within the specific context of the German Health Care System.

**Trial registration:**

ClinicalTrials.gov NCT02293252; date of registration: 10 November 2014

## Background

Chronic heart failure (CHF) is one of the most frequently diagnosed diseases causing disability and death in the Western hemisphere. It is characterized by a prevalence that increases with age [1]. In Germany, heart failure is the most common reason for hospitalization with about 396,000 cases in 2013 [2]. Direct medical costs related to heart failure account for 1–2 % of total health care expenditure [3].

In the majority of cases, home telemonitoring solutions in health care delivery to patients with CHF show advantages over usual care in terms of clinical outcomes. Several meta-analyses reveal that total mortality and number of hospitalizations tend to decrease, while patients’ quality of life improves [4–6]. Two subsequently published trials (Telemedical Interventional Management in Heart Failure trial (TIM-HF) [7], Telemonitoring in patients with Heart Failure trial (TELE-HF) [8]) show neutral findings in general. However, the health economic impact has not been clearly demonstrated so far [9]. A meta-analysis by Klersy et al. (2011) states that the difference in costs between remote patient monitoring and usual care ranges from Euro300 to Euro1000, favoring remote patient monitoring because of a lower hospitalization rate. Thus, direct costs for hospitalization were approximated by diagnosis-related group tariffs [10]. A more detailed evaluation of efficiency and economic feasibility could help to determine cost-effectiveness and to avoid misallocation of resources [11].

The CardioBBEAT trial was designed to assess the health economic impact of a dedicated home telemonitoring system for patients with CHF based on actual costs directly obtained from patients’ health care providers. The present report provides details on the outline of the study and an analysis of the study population’s baseline data.

## Methods

### Study design

CardioBBEAT represents a randomized, controlled, open, multicenter trial with two prospective study arms. Patients were recruited at ten study sites from five areas of varying economic status in Germany: namely, Berlin, Brandenburg, Bavaria, Hamburg and North Rhine-Westphalia. This diversity allows for investigating the impact of regional differences in medical care, with general medical care predominating in rural districts compared to predominantly specialist care in urban areas. Each site was responsible for recruiting as well as following-up on their patients. Specified information about inclusion and exclusion criteria is displayed in Table 1.

**Table 1:** Summary of inclusion and exclusion criteria at screening

Inclusion criteria
Confirmed diagnosis of CHF based on ESC guidelines
Symptoms corresponding to NYHA functional class II–IV
AHA classification stages C–D
LVEF ≤ 40 %
Age ≥ 18 years
Patient is discharged after being hospitalized for CHF within the last 12 months
Patient is able to understand the German language
Patient has sufficient eyesight to understand and follow the instructions communicated by Motiva®
Patient is willing and able to use the required hardware and software and to maintain a patient diary
Patient is residing within geographical reach of one of the ten telemonitoring centers in order to receive additional treatment if required as well as follow-up consultation
Patient gives informed consent regarding benefits and risks related to the trial, and to sign a participation agreement for the installation of the home telemonitoring system Motiva®
Exclusion criteria
Myocardial infarction within the past 4 weeks
Heart surgery or any coronary intervention within the past 8 weeks
Cardiogenic shock within the past 4 weeks
Intended cardiac surgery within the next 6 months or priority status on a waiting list for organ transplantation
Severe chronic and pulmonary illness with an immediate impact on the main outcome measures
Renal dysfunction requiring dialysis
Dementia or other severe cognitive impairment
Psychiatric disorders prohibiting a participation in the trial
Patient is discharged to or living in an older persons clinic or a nursing home
Patient is participating in another clinical trial

*AHA* American Heart Association, staging of heart failure, *CHF* chronic heart failure, *ESC* European Society of Cardiology, *LVEF* left ventricular ejection fraction *NYHA* New York Heart Association, classification of heart failure

The study has been conducted in accordance with the principles stated in the Declaration of Helsinki, the Good Clinical Practice (International Conference on Harmonization), and national as well as local regulations. The research protocol was approved by the responsible ethics committees (Table 2), and written informed consent was obtained from all patients prior to any study-related procedures. The study is monitored by an independent external institute to ensure that every participating site abides by the study protocol and to perform external quality control of the data.

**Table 2:** List of ethical bodies

Ethical body	Reference number
Ethik-Kommission für Forschungsfragen der Universität Bayreuth	O 1305 – HB/ID
Landesärztekammer Brandenburg, Ethik-Kommission	AS 94(a)/2009
Ethikkommission – Ethikausschuss 2 am Campus Virchow-Klinikum Berlin	EA2/084/09
Ethik-Kommission für Forschungsfragen der Universität Bayreuth	O 1305 – HB/ID
Ethik-Kommission für Forschungsfragen der Universität Bayreuth	O 1305 – HB/ID
Medizinische Fakultät der Universität Duisburg-Essen, Ethik-Kommission	10-4536
Ethik-Kommission der Bayerischen Landesärztekammer	7/11014
Universität Würzburg, Ethik-Kommission bei der Medizinischen Fakultät	128/11
Ethik-Kommission der Ärztekammer Hamburg	MC-141/11
Ethik-Kommission für Forschungsfragen der Universität Bayreuth	O 1305 – GB
Ärztekammer Nordrhein, Ethikkommission	2012451

### Setting

During the trial, all patients receive best medical treatment according to the current guidelines of the European Society of Cardiology (ESC) [12, 13].

Patients in the control group only receive best medical treatment as stated above, whereas patients in the intervention group additionally receive home telemonitoring-supported care that connects them to the participating care providers by individual guideline-compliant care plans using the telemedicine-system Motiva® (Philips Medical Systems GmbH, Hamburg, Germany).

Motiva® is an interactive bi-directional home telemonitoring system that provides remote monitoring, empowers patients to manage their disease state more effectively and enables physicians to keep in contact with the patient at home on a daily basis. Patients measure their vital signs (blood pressure, heart rate, and weight) every day and Motiva® transfers the data to the relevant telemonitoring center. In doing so, signals of decompensation regarding their heart function can be detected at an early stage and counteractive measures can be taken. In addition, patients receive information via Motiva®, i.e. coaching material, evaluations, reminders, and feedback regarding their health status as well as references to potentially necessary CHF treatment adaptations. If questionnaires reveal any problems, patients receive a phone call from the study site. Additionally, patients receive standardized questionnaires related to symptoms of cardiac decompensation, hypotension or hypertension or abnormal pulse rates. A call from the telemedicine center is made if patients gain more than 2 kg within 3 days, if their systolic blood pressure exceeds 140 mmHg or is lower than 90 mmHg, or their resting heart rate exceeds 80 bpm or is lower than 50 bpm.

A secured broadband connection (Digital Subscriber Line (DSL) or Universal Mobile Telecommunications System (UMTS)) and a set-top box turn the patient’s television into their center of personalized care protected by a patient-specific password. Thus, patients can transfer all information about their health status to their attending physician safely.

Motiva® is been provided by Philips Medical Systems GmbH, Hamburg, Germany. The telecommunication infrastructure to transfer patients’ data is made available by T-Systems International GmbH, Frankfurt, Germany. Both are provided without any obligations that could influence the study.

### Randomization

To assign patients to one of the two study arms, CardioBBEAT used a centralized stacked randomization technique. Patients at home who were managed in cardiologic practices were randomized patient-individually. Patients primarily managed by their general practitioner (GP), on the other hand, were cluster-randomized by their GP’s medical practices, to minimize carry-over effects and to keep the organizational effort manageable. The results of the randomization process with regard to the patients were displayed via the study’s electronic Case Report Form (eCRF) directly after inclusion into the trial. The study center in charge did inform the patients’ attending physicians whether their respective patients were enrolled in the trial and to which study arm they were assigned.

### Treatment patterns

After patients are discharged from inpatient care, their GPs or outpatient medical specialists will provide their ambulatory care. These physicians have access to individual patient care plans and are authorized to complete or modify them.

Subjects enrolled in the control group receive best medical treatment according to the current guidelines of the European Society of Cardiology (ESC). Subjects enrolled in the intervention group are additionally supported by the telemedicine system Motiva® installed at the patients’ home usually within 2 weeks.

All trial participants maintain a patient diary and are urged to document any health disturbances at least once a week, such as hospitalizations (date of hospitalization, reason for admission, and length of stay), consultations by any physician, and change in medication or dose rate as well as adverse effects. In addition, every patient has to participate in 3 trial-specific examinations (Table 3) at their relevant study site, that take place at the time of enrollment and after 6 as well as 12 months.

**Table 3:** Trial-specific examinations

Enrollment examination	
1. Patient briefing	
2. Written informed consent	
3. Verification of inclusion and exclusion criteria	
4. Demography	
Sex	
Date of birth	
Marital status	
Size and weight	
5. Hemodynamic parameters	
Heart rate	
Blood pressure (systolic/diastolic)	
6. Disease-related parameters	
Medical history	
CHF (date of diagnosis, aetiology, inpatient treatments, NYHA classification, AHA stadium)	
Comorbidities	
Medication	
Care plan compilation	
6 MWD	
7. Health-related quality of life assessment	
SF-36v2 (generic quality of life questionnaire)	
WHO-5 (generic quality of life questionnaire)	
KCCQ (disease-specific quality of life questionnaire)	
Follow-up and final examination	
1. Demography	
Weight	
2. Hemodynamic parameters	
Heart rate	
Blood pressure (systolic/diastolic)	
3. Disease-related parameters	
CHF (NYHA classification, AHA stadium)	
Newly occurring comorbidities	
Medication	
Hospitalizations or admissions to a nursing home	
Patient diary monitoring and validation of AE/SAE	
6 MWD	
4. Health-related quality of life assessment	
SF-36v2 (generic quality of life questionnaire)	
WHO-5 (generic quality of life questionnaire)	
KCCQ (disease-specific quality of life questionnaire)	
In case a patient dies during the trial	
Date of death	
Cause of death	

*6 MWD* 6-minute walking distance*, AE* adverse event, *AHA* American Heart Association, staging of heart failure, *ESC* European Society of Cardiology, *KCCQ* Kansas City Cardiomyopathy Questionnaire with 23 items for measuring disease-specific domains in CHF, *LVEF* left ventricular ejection fraction, *NYHA* New York Heart Association, classification of heart failure, *SAE* serious adverse event, *SF-36v2* short form health survey with 36 questions using norm-based scoring, *WHO-5* World Health Organization Five, well-being index

### Clinical outcome measures

The primary outcome measure to assess the benefit of home telemonitoring is the combined clinical endpoint “days alive and not in hospital nor inpatient care per potential days in study.” For deceased patients, the loss in lifetime is taken into account by setting the denominator to 360 days, for patients lost to follow-up, time to last contact is used. Secondary outcome measures are total mortality, number of inpatient treatments, length of stay in hospital or nursing home, functional state of health and health-related quality of life. These will be determined by the following parameters: days survived in the study, number of hospitalizations for any reason during the study (especially cardiac and heart failure-related reasons), number of days in hospital or nursing home per study month, generic (short form health survey with 36 questions using norm-based scoring (SF-36v2), World Health Organization Five, well-being index (WHO-5)) and disease-specific (Kansas City Cardiomyopathy Questionnaire, (KCCQ)) health-related quality of life as well as medical condition and capacity of each patient.

CardioBBEAT may also be able to differentiate between particular sub-groups (e.g. gender-specific, NYHA-specific, urban/rural, diabetes mellitus) while analyzing the effectiveness of the intervention. The expectation is that several patient groups can be identified which are particularly suited for home telemonitoring with regard to clinical and/or economic outcome.

### Cost data

CardioBBEAT aims to reflect the impact of home telemonitoring within an actual health care setting based on originally obtained cost data subdivided into cost of intervention, cost of inpatient and outpatient care, rehabilitation, nursing, and life-saving appliances. To this end, cost data are obtained from patients’ health insurance companies and later on validated using the records of the telemonitoring centers, GPs and medical specialists as well as patients’ diaries. Health insurance data will be obtained similarly for both, patients in the intervention group as well as the control group to avoid ascertainment bias.

All data are analyzed with appropriate statistical methods to determine the economic effectiveness of home telemonitoring for patients with CHF. Common approaches for the analysis of cost data such as *t* tests, analysis of covariance, bootstrap techniques or permutation tests will be compared regarding their feasibility, the validity of underlying assumptions and their stability and robustness in particular if missing values have to be taken into account.

Especially when analyzing cost data or determining the Incremental Cost-Effectiveness Ratio (ICER), assumptions are made regarding discount rate, utilities, projections or estimation of costs, which are based on uncertain hypotheses. To better understand the outcomes of these analyses, CardioBBEAT uses sensitivity analyses considering best-case and worst-case scenarios to demonstrate in which way the outcomes depend on these assumptions and how they affect their assessment.

### Statistical analysis

The primary endpoint ICER, consisting of the group’s difference in total cost and the combined clinical endpoint “days alive and not in hospital nor inpatient care per potential days in study”, is calculated with confidence intervals obtained by resampling methods. The comparative conventional endpoint “event-free survival” to measure the intervention’s effectiveness is evaluated using the Kaplan-Meier analysis and log rank tests. To incorporate possible repeated hospitalizations of a patient, additional analyses will be performed, e.g. comparison of quarterly data and recurrent event analysis.

Secondary outcome measures such as number of stays in hospital per quarter, health-related quality of life or time of survival are analyzed via permutation test, covariance analysis or log rank test as part of the Kaplan-Meier analysis.

Furthermore, the trial uses a cluster-randomization technique and, therefore, correlation effects can evolve due to the collective treatment of patients in a cardiology center, medical practice, or by a single study nurse. Such effects can result in incorrect *p* values. CardioBBEAT uses frailty and multi-level models to assess if and where such correlations occur. The magnitude of these correlations will be measured and the *p* values will be rectified.

Since at planning stage neither an established statistical method to directly compare costs and ICERs nor sufficient data to estimate the variability of cost estimates was available, the sample size was determined based on literature data. With respect to clinical endpoints, the figures were rather stable and converged to a minimum of 300 patients per group. Since the primary endpoint was expected to be mainly driven by clinical events, it was assumed that the sample size will also be sufficient for the continuously distributed ICER.

## Results

The study group comprised 621 patients, predominantly men. Four hundred and seventy-two (76 %) patients were treated by 449 GPs and 149 (24 %) were treated in 119 cardiologic practices. Three hundred and two patients were randomized into the intervention group and 319 patients into the control group. Ischemic cardiomyopathy was the leading cause of heart failure (59 %). Although randomly assigned, subjects of the control group were significantly more often in NYHA functional classes III or IV and exhibited peripheral edema or renal dysfunction, respectively, more frequently (Table 4). Additionally, the control and intervention group differed in heart rhythm disturbances (Table 5). No differences were detected regarding risk factor profile, comorbidities, echocardiographic parameters, especially left ventricular and diastolic diameter and ejection fraction, as well as functional test (6 MWD) results, medication and quality of life (Tables 4, 5, 6 and 7).

**Table 4:** Baseline characteristics of the CardioBBEAT study participants

Characteristic	All patients *n* = 621	Usual care *n* = 319 (51 %)	Monitoring *n* = 302 (49 %)	*p* value
Demographic profile				
Age (years)				
mean ± SD	63.0 ± 11.5	63.5 ± 11.4	62.5 ± 11.6	0.303
median (IQR)	65 (55–72)	65 (55–73)	64 (54–72)	
Male sex, *n* (%)	544 (88)	280 (88)	264 (87)	0.990
Living alone, *n* (%)	159 (26)	77 (24)	82 (27)	0.442
Education (years) – number of patients (% valid)	607 (98)	311 (98)	296 (98)	
mean ± SD	12 ± 3	12 ± 3	12 ± 3	0.803
median (IQR)	11 (10–13)	11 (10–13)	12 (10–13)	
Causes of heart failure, *n* (%)				0.797
Ischemic CM	363 (59)	185 (58)	178 (59)	
Non-ischemic CM	258 (42)	134 (42)	124 (41)	
NYHA class, *n* (%)				0.086
II	430 (69)	209 (66)	221 (73)	
III	186 (30)	108 (34)	78 (26)	
IV	5 (1)	2 (1)	3 (1)	
NYHA class III–IV, *n* (%)	191 (31)	110 (35)	81 (27)	0.048
Peripheral edema, *n* (%)	131 (21)	83 (26)	48 (16)	0.003
Comorbidities, *n* (%)				
Stroke/TIA	41 (7)	21 (7)	20 (7)	1.000
PAD	56 (9)	24 (8)	32 (11)	0.232
COPD	87 (14)	48 (15)	39 (13)	0.516
Sleep apnea	45 (7)	25 (8)	20 (7)	0.668
Renal dysfunction (GFR ≤ 60 ml/min)	148 (24)	87 (27)	61 (20)	0.048
Depression	51 (8)	24 (8)	27 (9)	0.619
Resuscitation	81 (13)	37 (12)	44 (15)	0.327
Risk factor profile				
BMI (kg/m^2^) – number of patients (% valid)	619 (100)	318 (100)	301 (100)	
mean ± SD	28 ± 5	28 ± 5	28 ± 5	0.529
median (IQR)	28 (25–31)	28 (25–32)	27 (25–31)	
History of smoking – number/total number (%)	435/620 (70)	221/319 (69)	214/301 (71)	0.684
Diabetes mellitus, *n* (%)	219 (35)	103 (32)	116 (38)	0.131
Hypertension, *n* (%)	533 (86)	269 (84)	264 (87)	0.323

*BMI* body mass index*, CM* cardiomyopathy, *COPD* chronic obstructive pulmonary disease, *GFR* glomerular filtration rate, *IQR* interquartile range, *NYHA* New York Heart Association, classification of heart failure, *PAD* peripheral arterial disease, *TIA* transient ischemic attack

**Table 5:** Baseline characteristics of the CardioBBEAT study participants **-** Diagnostic

Characteristic	All patients *n* = 621	Usual care *n* = 319 (51 %)	Monitoring *n* = 302 (49 %)	*p* value
ECG				
Heart rate (1/min) – number of patients (% valid)	616 (99)	315 (99)	301 (100)	
mean ± SD	72 ± 14	72 ± 14	72 ± 13	0.862
median (IQR)	71 (63–81)	71 (63–82)	71 (62–80)	
Heart rhythm – number/total number (%)				0.031
Sinus rhythm	379/619 (61)	185/318 (58)	194/301 (65)	
Atrial fibrillation	78/619 (13)	46/318 (15)	32/301 (11)	
Pacemaker ECG	154/619 (25)	86/318 (27)	68/301 (23)	
Other	8/619 (1)	1/318 (0)	7/301 (2)	
Conduction disorder – number/total number (%)				
LBBB	145/543 (27)	69/272 (25)	76/271 (28)	0.543
RBBB	49/544 (9)	28/273 (10)	21/271 (8)	0.383
QRS duration (ms) – number of patients (% valid)	568 (92)	288 (90)	280 (93)	
mean ± SD	123 ± 33	125 ± 34	121 ± 32	0.151
median (IQR)	110 (100–144)	116 (100–150)	110 (100–140)	
2D echocardiography				
LVEDD (mm) – number of patients (% valid)	584 (94)	297 (93)	287 (95)	
mean (mm) ± SD	62 ± 9	62 ± 9	63 ± 9	0.580
median (IQR)	62 (57–68)	62 (57–68)	62 (57–68)	
LVEF (%) – number of patients (% valid)	619 (100)	317 (100)	302 (100)	
mean ± SD	30 ± 7	31 ± 7	30 ± 8	0.580
median (IQR)	31 (25–37)	31 (25–37)	30 (25–36)	
Mitral insufficiency – number/total number (%)				0.319
none	102/614 (17)	55/315 (18)	47/299 (16)	
mild	370/614 (60)	180/315 (57)	190/299 (64)	
moderate	121/614 (20)	70/315 (22)	51/299 (17)	
severe	21/614 (3)	10/315 (3)	11/299 (4)	
6-minute walk test – number of patients (% valid)	559 (90)	284 (89)	275 (91)	
mean (m) ± SD	375 ± 132	376 ± 132	374 ± 131	0.804
median (IQR)	400 (300–458)	404 (300–455)	400 (300–460)	

*2D* two-dimensional, *ECG* electrocardiogram, *IQR* interquartile range, *LBBB* left bundle branch block,*LVEDD* left ventricular end-diastolic dimension, *LVEF* left ventricular ejection fraction, *QRS* combination of three of the graphical deflections seen on a typical ECG, *RBBB* right bundle branch block

**Table 6:** Baseline characteristics of the CardioBBEAT study participants **-** Therapy

Characteristic	All patients *n* = 621	Usual care *n* = 319 (51 %)	Monitoring *n* = 302 (49 %)	*p* value
Medication				
ACE inhibitor/ARB, *n* (%)	577 (93)	297 (93)	280 (93)	0.974
Beta blocker – number/total number (%)	591/620 (95)	303/319 (95)	288/301 (96)	0.826
MR antagonist, *n* (%)	439 (71)	232 (73)	207 (69)	0.291
Diuretics, *n* (%)	506 (82)	255 (80)	251 (83)	0.360
Glycosides, *n* (%)	95 (15)	50 (16)	45 (15)	0.876
Amiodarone, *n* (%)	80 (13)	48 (15)	32 (11)	0.125
Anticoagulation, *n* (%)				
Vitamin K antagonist	231 (37)	122 (38)	109 (36)	0.637
Other	42 (7)	24 (8)	18 (6)	0.538
Devices, *n* (%)				
Pacemaker	101 (16)	61 (19)	40 (13)	0.061
ICD				0.280
with monitoring	63 (10)	31 (10)	32 (11)	
without monitoring	242 (39)	134 (42)	108 (36)	
CRT-D	89 (14)	40 (13)	49 (16)	0.232

*ACE* angiotensin converting enzyme, *ARB* angiotensin receptor blocker, *CRT-D* cardiac resynchronization therapy combined with defibrillation *ICD* implantable cardioverter defibrillator, *MR* mineralocorticoid receptor

**Table 7:** Baseline characteristics of the CardioBBEAT study participants – Quality of life

Characteristic	All patients *n* = 621	Usual care *n* = 319 (51 %)	Monitoring *n* = 302 (49 %)	*p* value
SF-36v2				
Physical comp. sum – number of patients (% valid)	581 (94)	299 (94)	282 (93)	
mean ± SD	39 ± 10	39 ± 10	38 ± 10	0.458
median (IQR)	38 (32–46)	38 (32–46)	38 (32–45)	
Mental comp. sum – number of patients (% valid)	581 (94)	299 (94)	282 (93)	
mean ± SD	45 ± 13	45 ± 12	44 ± 13	0.239
median (IQR)	46 (35–56)	46 (36–55)	45 (34–56)	
Physical functioning – number of patients (% valid)	591 (95)	304 (95)	287 (95)	
mean ± SD	51 ± 27	52 ± 27	50 ± 27	0.445
median (IQR)	50 (30–75)	50 (30–75)	50 (30–70)	
WHO-5				
Score – number of patients (% valid)	586 (94)	301 (94)	285 (94)	
mean ± SD	55 ± 25	55 ± 25	54 ± 25	0.588
median (IQR)	56 (36–76)	56 (36–76)	56 (32–76)	
KCCQ				
Overall sum – number of patients (% valid)	591 (95)	305 (96)	286 (95)	
mean ± SD	59 ± 24	59 ± 24	60 ± 23	0.574
median (IQR)	61 (42–80)	60 (42–80)	62 (42–80)	
Clinical sum – number of patients (% valid)	591 (95)	305 (96)	286 (95)	
mean ± SD	63 ± 25	63 ± 26	64 ± 24	0.409
median (IQR)	67 (45–85)	67 (44–85)	69 (49–84)	

*Comp* component, *IQR* interquartile range, *KCCQ* Kansas City cardiomyopathy questionnaire with 23 items for measuring disease-specific domains in CHF, *SF-36v2* short form health survey with 36 questions using norm-based scoring, *sum* summary, *WHO-5* World Health Organization Five, well-being index

In comparison with recently published German trials (TIM-HF [7], Interdisciplinary Network for Heart failure study (INH) [14]) the CardioBBEAT target population was slightly younger and comprised more male patients with fewer in NYHA classes III and IV. Nevertheless, all patients were either categorized in AHA stages C or D and every fifth patient of the given cohort was diagnosed with peripheral edema; this suggests that the study population had a comparable degree of heart failure. Regarding comorbidities and risk factor profile, the study population was very similar, particularly for diabetes or renal dysfunction. Remarkably, it revealed a left bundle branch block in approximately 25 % of the population, a rhythm disorder with a known worse prognosis [15]. Furthermore, detailed information on prognostic relevant therapies was documented. Besides a high proportion of guideline-based pharmacotherapy including beta blockers and ACE inhibitors/ARB inhibitors, the study population was treated with mineralocorticoid receptor blockers in 71 % of all cases. A cardioverter-defibrillator or a resynchronization system was implanted in two thirds of our patients (comparable to TIM-HF study [7]) with a considerable prognostic impact on primary and secondary endpoints. Table 8 compares the most important baseline characteristics with TIM-HF [7] and INH [14].

**Table 8:** Compared baseline characteristics of the CardioBBEAT, TIM-HF and INH study participants

Variable	CardioBBEAT		TIM-HF		INH	
	Usual care	Monitoring	Usual care	Monitoring	Usual care	Monitoring
	(*n* = 319)	(*n* = 302)	(*n* = 356)	(*n* = 354)	(*n* = 363)	(*n* = 352)
Demographic profile						
Age (years)	63.5 ± 11.4	62.5 ± 11.6	66.9 ± 10.5	66.9 ± 10.8	69.4 ± 11.5	67.7 ± 12.8
Male sex (%)	88	87	82	81	71	71
Living alone (%)	24	27	22	21	35	30
Clinical profile						
NYHA class (%)						
I	0	0	0	0	2	3
II	65	73	51	50	62	54
III	34	26	49	50	31	40
IV	1	1	0	0	5	3
Risk factor profile						
BMI (kg/m^2^)	28 ± 5	28 ± 5	28 ± 5	28 ± 5	n.a.	n.a.
Diabetes mellitus (%)	32	38	39	40	36	36
Hypertension (%)	84	87	66	68	77	72
Diagnostic						
Heart rate (1/min)	72 ± 14	72 ± 13	71 ± 13	71 ± 13	80 ± 18	80 ± 20
LVEF (%)	31 ± 7	30 ± 8	27 ± 6	27 ± 6	30 ± 8	30 ± 8
Medication (%)						
ACE inhibitor/ARB	93	93	97	94	87	89
Beta blocker	95	96	93	92	79	81
Diuretics	80	83	94	94	86	90

*ACE* angiotensin converting enzyme*, ARB* angiotensin receptor blocker, *BMI* body mass index*, LVEF* left ventricular ejection fraction, *n.a.* not available*, NYHA* New York Heart Association, classification of heart failure

## Discussion

Several recent randomized controlled trials (RCTs), including TIM-HF [7, 16] and INH [14], have proven the positive clinical effects of home telemonitoring on several groups of patients diagnosed with CHF. The meta-analyses of Clark et al. [4], Klersy et al. [5] and Inglis et al. [6] also showed its potential to improve several clinical outcomes such as quality of life. However, many results were not statistically significant mostly due to the fact that effects were too small despite adequately sized studies.

Another meta-analysis by Klersy et al. [10] focused on the economic impact of remote patient monitoring. It showed that management of HF patients by remote monitoring is cost-saving due to a substantial reduction in health care resource utilization, mostly driven by a reduction in the number of HF hospitalizations. However, cost data in this meta-analysis was estimated using 3 diagnosis-related group reimbursements (minimum, median, maximum over countries) and 3 different incidence rates and their lower and upper 95 % CI (confidence interval). These facts reflect the requirement for additional study-derived and reliable evidence based on originally obtained cost data unlike previously negotiated prices.

In CardioBBEAT, the follow-up care of patients was more diverse than expected: the number of participating practices was higher whereas the number of patients per practice was lower than expected, resulting in an incomplete use of the random blocks implemented in the eCRF and slightly unequal sample sizes between the random groups. However, slight differences in group sizes are of no concern with respect to unbiasedness of results.

At first glance, an imbalance in baseline variables stands out. But, even with perfect randomization the expected number of statistically significant differences between baseline variables is 5 % or 2.45 of the 49 baseline comparisons in Tables 4, 5, 6 and 7, on average. In case of independence of the baseline variables, the observed number of significances will thus follow a binomial distribution with *p* = 0.05. In CardioBBEAT, 4 out of 49 baseline comparisons (8.2 %) were significant. In case of independence, 4 or more significant comparisons would occur in 23 % of the cases even in perfect randomization. The observed baseline differences could thus well be a play of chance. However, NYHA functional class, peripheral edema, heart rhythm, and renal dysfunction were clinically highly relevant variables that might bias the conclusions even if evoked by chance. Therefore, the statistical analysis plan was extended to include adjusted analyses with respect to the baseline imbalances.

## Conclusions

CardioBBEAT is a RCT that adds a comprehensive cost assessment to the clinical component of the study including actual costs generated by patients, health services and health products. The corresponding data have been obtained directly from patients’ health insurances including statutory sickness funds and private insurances. This will provide more reliable information about the cost-effectiveness of home telemonitoring in CHF patients based on the actual health care setting. CardioBBEAT may also be able to differentiate between particular sub-groups (gender-specific, NYHA-specific, urban/rural, diabetes mellitus) while analyzing the effectiveness of the intervention. The expectation is that important patient groups, which are better suited for the input of telemedicine with regard to the clinical and/or economic outcome, can be identified. The study results, reflecting a guideline-compliant, highly accurate treatment of the whole CardioBBEAT study population shown above, will significantly contribute to the existing data basis on home telemonitoring in CHF. Therefore, it adds to informed political and economic decision-making within the specific context of the German Health Care System.

### Individual gratitude

**Table:** 

Study center home telemonitoring teams		
German Heart Institute Berlin		
Fleck Eckart, Prof. Dr.	Furundzija Vesna, Dr.	Götze Stephan , PD Dr.
Roser Mattias, Dr.	Ahmed-Taner Funda, Dr.	Weinkopf Katharina
Gültekin Nicole	Post Monica	
Klinik am See Rüdersdorf		
Völler Heinz, Prof. Dr.	Belozerov Sergej, Dr.	Michely Beate, Dr.
Hartwig-Zaidan Ines	Stolze Kirsten	Salzwedel Annett
University Hospital Essen		
Neumann Till, Prof. Dr.	Krings Peter, Dr.	
Comprehensive Heart Failure Center Würzburg		
Störk Stefan, Prof. Dr.	Angermann Christiane, Prof. Dr.	Reichert Clemens, Dr.
Schmidt Maximilian, Dr.	Menhofer Dominik, Dr.	Hartner Gabriele
Schupfner Elisabeth		
Contilia Heart and Vascular Center Essen		
Bruder Oliver, PD Dr.	Blank Elisabeth	Grosch Bernhard, Dr.
Seifert Vanessa, Dr.	Waidelich Lioba, Dr.	Keinhorst Jens
Reuter Vanessa	Steffen Melanie	
Munich Municipal Hospital Bogenhausen		
Leber Alexander, PD Dr.	Antoni Diethmar, Dr.	Kloos Patrick, Dr.
Landwehr Peter, Dr.	Tomelden June	
Vivantes Medical Center Berlin Neukölln		
Darius Harald, Prof. Dr.	Meincke Carsten, Dr.	Kirchner Aenn
Maselli Astrid		
Jewish Hospital Berlin		
Graf Kristof, Prof. Dr.	Bolay Miriam, Dr.	Pfürtner Mona, Dr.
Fischer Lidia		
University Heart Center Hamburg-Eppendorf		
Patten-Hamel Monica, PD Dr.	Molz Simon, Dr.	Sinning Christoph, Dr.
de Boer Imkje	Hermes Monika	Kupper-Schmidt Claudia
Hospitals Essen Süd		
Koslowski Bernd, Dr.	Kemper Nicole	Reintges Nicole
Technology partners		
Philips Medical Systems Boeblingen GmbH		
Brüge Armin, MBA, Dipl.-Ing.	Bui Nhat Kha, Certified Engineer, Dipl. Can. Theol.	Goldbach Udo, Dipl.-Ing.
Richter Wolfgang, Dr.	Sarantos Melanie	Westerteicher, Christoph, Dipl.-Ing.
T-Systems International GmbH		
Bruns Uta	Cech Martin, Dipl.-Ing.	Foth Gerd, Dipl.-Ing.
Hauptmann Imke		
Study and project management		
Institute of Health Care Management and Health Sciences		
Nagels Klaus, Prof. Dr. Dr.	Nagel Eckhard, Prof. Dr. mult.	Wohlgemuth Walter, Prof. Dr. Dr.
Dittmar Ronny, Dr.	Hofmann Reiner	Bindl Dominik
Department of Medical Biometry and Epidemiology		
Wegscheider Karl, Prof. Dr.	Balzer Klaus	Treszl Andras, Dr.
Vettorazzi Eik		
Monitoring		
Clinical Trial Center North		
Freese Ralf, Dr.	Papavlassopoulos Martin, Dr.	Henkes Liliane, Dr.
Borregaard Saskia, Dr.		
Development of cost data set		
AOK Nordost		
Kornek Stefanie	Schönfelder Moritz	
DAK-Gesundheit		
Wobbe Stefanie		
TK		
Kettlitz Mandy	Petereit Frank	

## Abbreviations

ACE: angiotensin converting enzyme
AE: adverse event
AHA: American Heart Association, staging of heart failure
ARB: angiotensin receptor blocker
BMI: body mass index
CHF: chronic heart failure
CI: confidence interval
CM: cardiomyopathy
COPD: chronic obstructive pulmonary disease
CRT-D: cardiac resynchronization therapy combined with defibrillation
DSL: Digital Subscriber Line (broadband connection)
ECG: electrocardiogram
eCRF: electronic Case Report Form
ESC: European Society of Cardiology
GFR: glomerular filtration rate
GP: general practitioner
HF: heart failure
ICD: implantable cardioverter defibrillator
ICER: Incremental Cost-Effectiveness Ratio
INH: Interdisciplinary Network for Heart failure study
KCCQ: Kansas City Cardiomyopathy Questionnaire with 23 items for measuring disease-specific domains in CHF
LBBB: left bundle branch block
LVEDD: left ventricular end-diastolic dimension
LVEF: left ventricular ejection fraction
MR: mineralocorticoid receptor
MWD: minute walking distance
n.a.: not available
NYHA: New York Heart Association, classification of heart failure
PAD: peripheral arterial disease
RBBB: right bundle branch block
RCT: randomized controlled trial
SAE: serious adverse event
SF-36v2: short form health survey with 36 questions using norm-based scoring
TELE-HF: Telemonitoring in patients with Heart Failure trial
TIA: transient ischemic attack
TIM-HF: Telemedical Interventional Management in Heart Failure trial
UMTS: Universal Mobile Telecommunications System (mobile cellular system)
WHO-5: World Health Organization Five, well-being index
